# Supplementary material for: Effect of β-Glucan Supplementation on Growth Performance and Intestinal Epithelium Functions in Weaned Pigs Challenged by Enterotoxigenic Escherichia coli
Source: Antibiotics (Basel). 2022 Apr 13;11(4):519. doi: 10.3390/antibiotics11040519 (PMC9029716; doi:10.3390/antibiotics11040519)
Supplement: Supplementary file 1 [file antibiotics-11-00519-s001.zip › antibiotics-1659862-supplementary.pdf]

Table S1. Primers sequences used for quantitative RT-PCR

Gene	Primer sequence (5' –3')	Annealing temperature (°C)	Product size (bp)
β-Actin	F: TGGAACGGTGAAGGTGACAGC<br>R: GCTTTTGGGAAGGCAGGGACT	60	177
SGLT-1	F: CCACTTTCCCTATAAAACCTCAC<br>R: CTCCATCAAACTTCCATCCTCAG	60	151
GLUT-2	F: CCTGCTTGGTCTATCTGCTGTG<br>R: TTGATGCTTCTTCCCTTTCTTT	60	156
CAT-1	F: TGCCCATACTTCCCGTCC<br>R: GGTCCAGGTTACCGTCAG	60	192
LAT-1	F: GCCCATTGTCACCATCATC<br>R: GAGCCCACAAAGAAAAGC	60	216
FATP-1	F: GGAGTAGAGGGCAAAGCAGG<br>R: AGGTCTGGCGTGGGTCAAAG	60	208
FATP-4	F: TTCATCAAGACGGTCAGGCG<br>R: AGACGGTGGCAGCGAATAAG	60	133
ZO-1	F: CAGCCCCCGTACATGGAGA<br>R: GCGCAGACGGTGTTCATAGTT	60	114
Occludin	F: CTACTCGTCCAACGGGAAAG<br>R: ACGCCTCCAAGTTACCACTG	60	158
Claudin-1	F: GCCACAGCAAGGTATGGTAAC<br>R: AGTAGGGCACCTCCCAGAAG	60	140
MUC2	GGTCATGCTGGAGCTGGACAGT<br>TGCCTCCTCGGGGTCGTCAC	59	181
Total bacteria	F: ACTCCTACGGGAGGCAGCAG<br>R: ATTACCGCGGCTGCTGG	60	200
Lactobacillus	F: GAGGCAGCAGTAGGGAATCTTC<br>R: CAACAGTTACTCTGACACCCGTTCTTC<br>P: AAGAAGGGTTTCGGCTCGTAAAACTCTGTT	60	126
Escherichia coli	F: CATGCCGCGTGTATGAAGAA<br>R: CGGGTAACGTCAATGAGCAAA<br>P: AGGTATTAACTTTACTCCCTTCCTC	60	96
Bifidobacterium	F: CGCGTCCGGTGTGAAAG<br>R: CTTCCCGATATCTACACATTCCA	60	121

	P: ATTCCACCGTTACACCGGGAA		
	F: GCAACGAGCGCAACCCTTGA		
Bacillus	R: TCATCCCCACCTTCCTCCGGT	60	92
	P: CGGTTTGTCACCGGCAGTCACCT		

ZO-1, zonula occludens-1; FATP1, Fatty acid transport protein-1; FATP4, Fatty acid transportprotein-4; LAT1, L-type amino acid transporter-1; CAT1, cationic amino acid transporter-1; MUC2, mucin-2; SGLT1,sodium glucose transport protein-1;
